# Supplementary material for: Early indicators of tidal ecosystem shifts in estuaries
Source: Nat Commun. 2023 Apr 6;14:1911. doi: 10.1038/s41467-023-37444-6 (PMC10079839; doi:10.1038/s41467-023-37444-6)
Supplement: Supplementary file 1 — Supplementary Information [file 41467_2023_37444_MOESM1_ESM.pdf]

Supplementary Information

Early indicators of tidal ecosystem shifts in estuaries

Gregory S. Fivash*, Stijn Temmerman, Maarten G. Kleinhans, Maike Heuner, Tjisse van der Heide, Tjeerd J. Bouma

*greg.fivash@nioz.nl

Supplementary Table 1: NDVI thresholds used to classify vegetation in the Western Scheldt and Humber estuaries. The vegetated areas of the Elbe were classified using existing biotope/vegetation maps.

Estuary	Year	NDVI-threshold
Western Scheldt	2004	0.070
Western Scheldt	2008	0.065
Western Scheldt	2010	0.095
Western Scheldt	2011	0.170
Western Scheldt	2012	0.075
Western Scheldt	2015	0.145
Western Scheldt	2016	0.090
Western Scheldt	2018	0.065
Western Scheldt	2020	0.140
Humber	2011	0.0600
Humber	2012	-0.045
Humber	2013	-0.340
Humber	2014	-0.290
Humber	2015	-0.195
Humber	2016	0.027
Humber	2018	-0.255

Supplementary Table 2: Linear regression fits of the regressions depicted in Figure 2a and Supplementary Figure 1. These include the change over time, in each estuary, of (1) tidal elevation above neap high water level, and (2) vegetated area.

Estuary	Response variable	Avg. change	SE	n	F	df	R^2	p
Western Scheldt	Elevation (cm yr^{-1})	+ 1.44	± 0.10	16	226	14	0.94	5.0 $x10^{-10}$
Western Scheldt	Skipped inundations (% yr^{-1})	+ 0.32	± 0.02	16	261	14	0.95	$1.9x10^{-10}$
Western Scheldt	Vegetated area (% yr^{-1})	+ 0.40	± 0.06	9	46	7	0.85	0.00025
Western Scheldt	Vegetated area (ha yr^{-1})	+ 37.91	± 5.56	9	46	7	0.85	0.00025
Humber	Elevation (cm yr^{-1})	+ 3.23	± 0.21	11	233	9	0.96	9.7 $x10^{-10}$
Humber	Skipped inundations (% yr^{-1})	+ 1.31	± 0.08	11	304	9	0.97	3.0 $x10^{-10}$
Humber	Vegetated area (% yr^{-1})	+ 2.42	± 0.43	7	32	5	0.84	0.0024
Humber	Vegetated area (ha yr^{-1})	+ 15.98	± 2.83	7	32	5	0.84	0.0024
Elbe	Elevation (cm yr^{-1})	+ 1.42	± 0.09	3	275	1	0.99	0.038
Elbe	Skipped inundations (% yr^{-1})	+ 0.15	± 0.0001	3	103k	1	1	0.0006
Elbe	Vegetated area (% yr^{-1})	+ 0.26	± 0.06	4	21	2	0.87	0.044
Elbe	Vegetated area (ha yr^{-1})	+ 17.30	3.74	4	21	2	0.87	0.044

Supplementary Note 1: Public data portals

Western Scheldt
Dutch geospatial data portal (Rijkwaterstaat geodata):
https://www.rijkswaterstaat.nl/apps/geoservices/geodata/dmc/?C=N;O=A
Dutch water level time series data (Rijkwaterstaat Waterinfo):
https://waterinfo.rws.nl/

Humber
British geospatial data portal (Defra Survey):
https://environment.data.gov.uk/DefraDataDownload/?Mode=survey
British water level time series data portal (NTSLF):
https://ntslf.org/data/uk-network-real-time

Elbe
Both geospatial data and water level time series were provided by the German Federal Waterways and Shipping administration (WSV) and are available via the following online data portals:
https://www.kuestendaten.de/Tideelbe/DE/Service/Kartentool/Kartentool_node.html
https://www.kuestendaten.de/DE/Services/Messreihen_Dateien_Download/Download_Zeitreihen_node.html

Supplementary Figure 1. **The change in the vegetated area of the Humber, Western Scheldt, and Elbe over the years of data avilability fit to linear regressions.** The fits of each regression are detailed in Supplementary Table 2. On the left y-axis the vegetated area is calculated in hectares, while on the right vegetation is calcualted as percent cover of the intertidal within the sampled regions of the estuaries. Each estuary has experienced a considerable increase in the total vegetated intertidal area within this period. Source data are provided as a Source Data file.

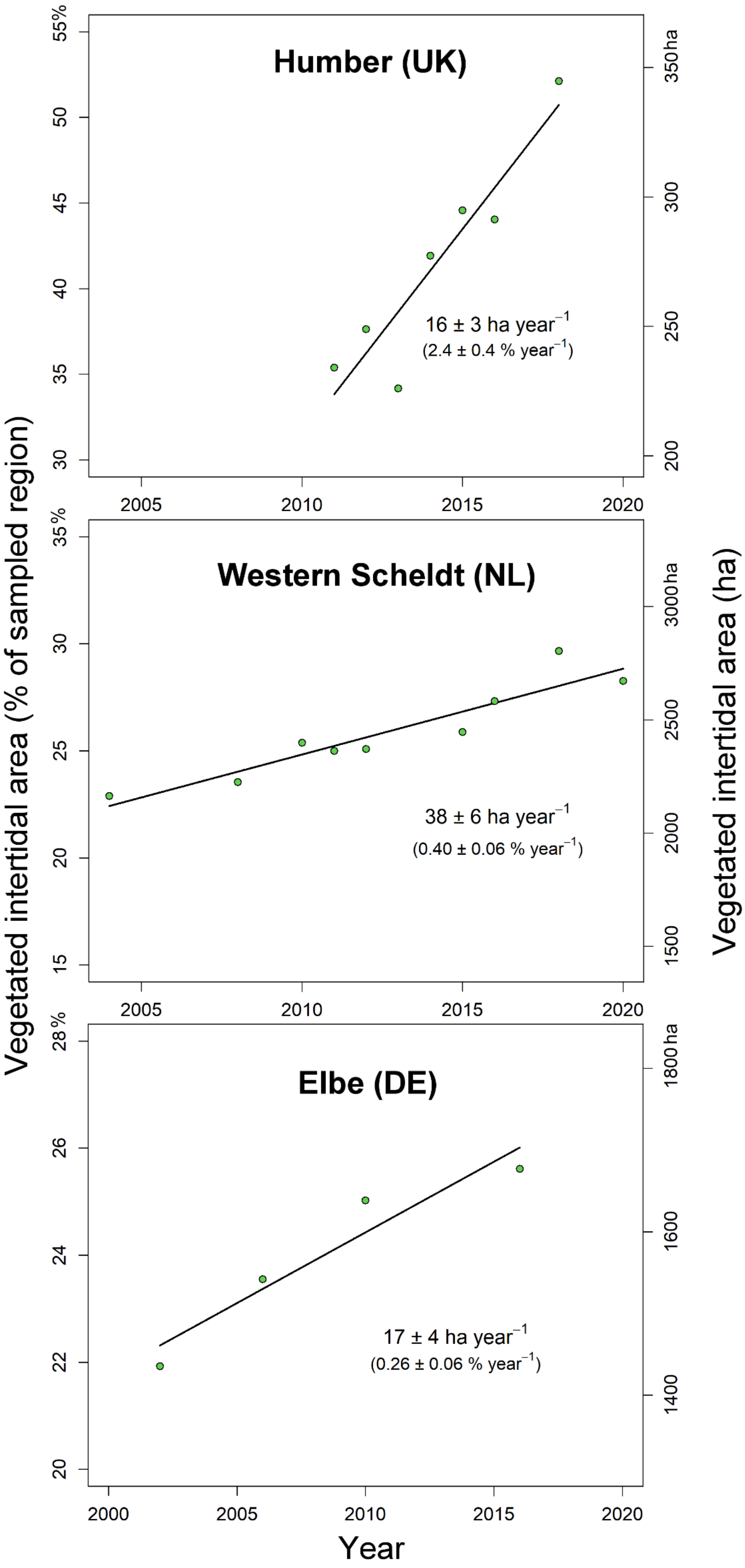
Humber (UK)
16 ± 3 ha year⁻¹
(2.4 ± 0.4 % year⁻¹)
Western Scheldt (NL)
38 ± 6 ha year⁻¹
(0.40 ± 0.06 % year⁻¹)
Elbe (DE)
17 ± 4 ha year⁻¹
(0.26 ± 0.06 % year⁻¹)
Vegetated intertidal area (% of sampled region)
Vegetated intertidal area (ha)
Year

Supplementary Figure 2. **A schematic illustration explaining the calculation and utility of the 'tidal inundations skipped (%)' metric.** Panel A showcases the large variation in high tide levels in a tidal signal over a year. The red lines, appearing again in each panel, demark the position of 1, 10, 50, and 90 % tidal inundations skipped. In Panel B, the non-linear relationship between elevation and skipped tidal inundations is displayed. In Panel C, the probability of vegetation presence is displayed against skipped tidal inundations. The tide data displayed here (Panels A & B) was calculated using data from the Western Scheldt (2004 -2020). Note the utility of this metric because at 0 % skipped inundations, the probability of vegetation presence is also zero. Due to the non-linear relationship between elevation and skipped tidal inundations, and again between skipped inundations and the probability of vegetation, linear annual increases in intertidal elevation can continue for a long time without increasing the vegetated area (in this case in the elevation range below 150 cm NAP), only to suddenly begin initiating vegetation expansion, once the elevation reaches a critical range (here in the range between 150 – 300 cm NAP). Note that the relationship between elevation and tidal inundations will vary between estuaries according to the tidal amplitude and the local variation in high tide levels, as seen the bottom panel. Source data are provided as a Source Data file.

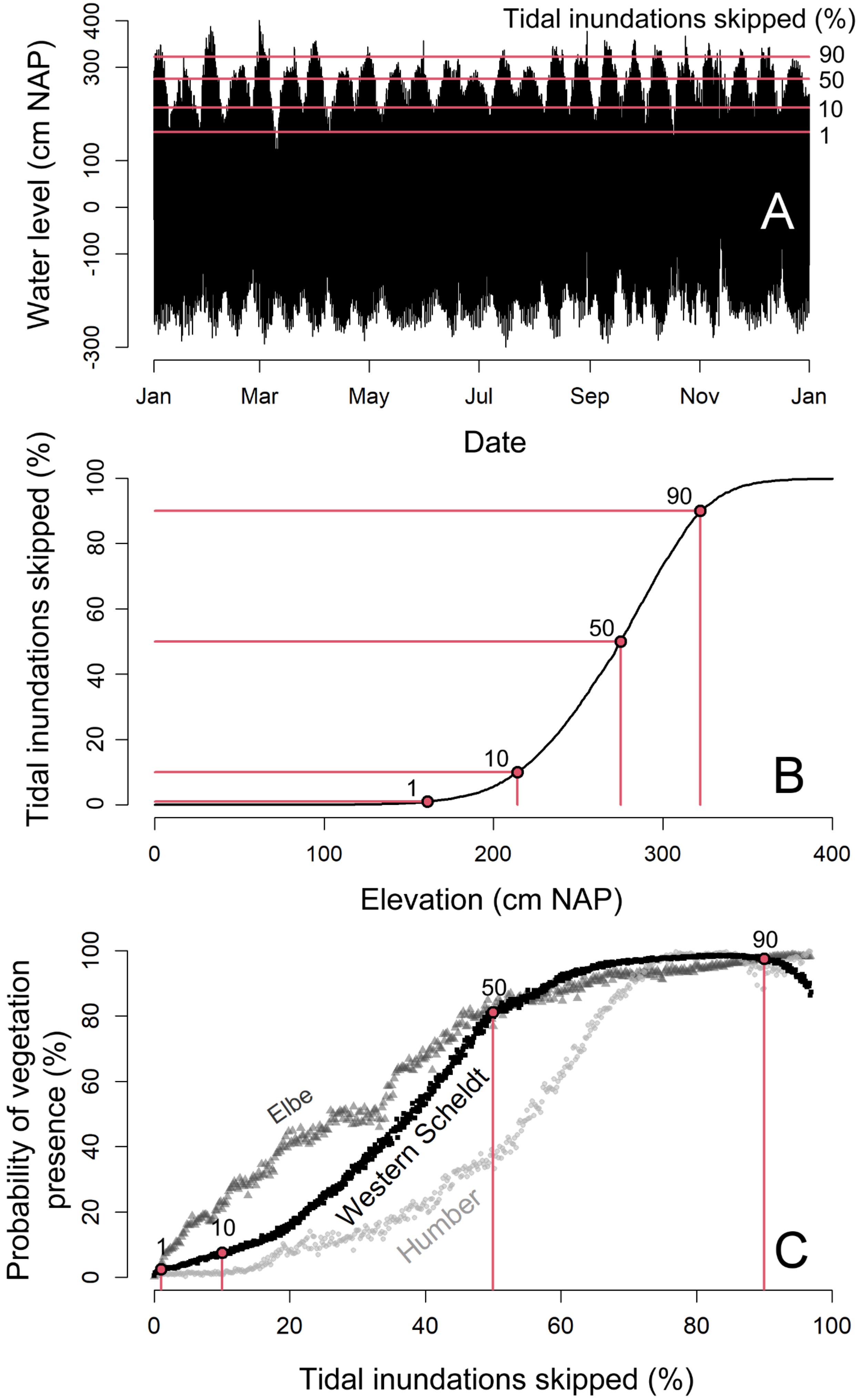
Tidal inundations skipped (%)
90
50
10
1
A
Water level (cm NAP)
400
300
100
0
-100
-300
Jan
Mar
May
Jul
Sep
Nov
Jan
Date
B
Tidal inundations skipped (%)
100
80
60
40
20
0
200
300
400
Elevation (cm NAP)
C
Probability of vegetation presence (%)
Elbe
Western Scheldt
Humber
Tidal inundations skipped (%)

Supplementary Figure 3. **The effects of tidal flat slope and elevation on micro-topographic intensity are displayed in four panels, considering all three studied estuaries.** In Panel A, the interactive effects of tidal position and slope on micro-topographic intensity are explored over a 2D field. Here darker colors represent on average more intense micro-topographic patterning. Panel B shows the number of observations at each cell in the field (cells supported by less than 30 observations have been removed). Note the strong correlation between high position in the intertidal and low slope tidal flats, indicated by the distribution of observations across this frame. Panels C and D display the effects of tidal inundation (C) and slope (D) independently. Here, points represent the average of a binned group and error bars show standard error (the exact number of replicate measurements within each binned group (n) for each mean can be found in the source data. Source data are provided as a Source Data file). The line in panel C shows the fit of a linear regression after both variables have been log-transformed. These panels show that micro-topography is stronger when higher in the tidal frame on very shallow slopes. However, panel A shows that micro-topgraphy only occurs in the upper intertidal within low slope regions (slopes less than 0.2 - 0.3 degrees), indicating that slope may be the predominant precursor of pattern formation.

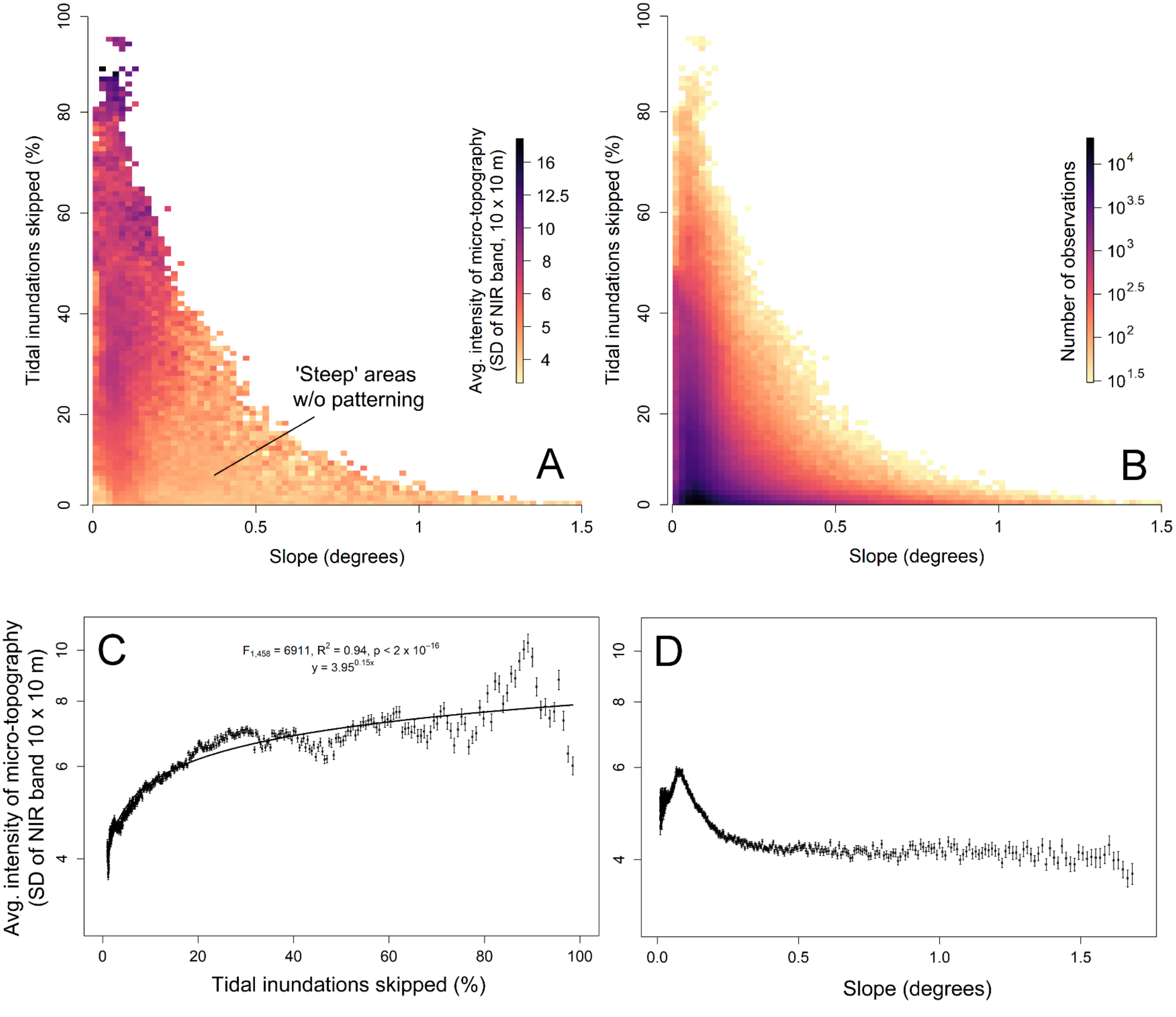

A
B
C
D
Tidal inundations skipped (%)
Slope (degrees)
'Steep' areas
w/o patterning
Avg. intensity of micro-topography
(SD of NIR band, 10 x 10 m)
Number of observations
Avg. intensity of micro-topography
(SD of NIR band 10 x 10 m)
F1,458 = 6911, R2 = 0.94, p < 2 x 10−16
y = 3.95^0.15x

***Supplementary Figure 4*. A showcase of a typical accreting upper intertidal flat that experiences pattern formation and subsequent vegetation establishment.** Here we seen the development of the tidal flats at Zuidgors and Baarland in the Western Scheldt between 2004 and 2022. In the topmost panels we can see the change in (A) elevation and (B) micro-topographic pattern intensity (SD of NIR-band) between 2004 and 2020. The bottomright panel (C) displays the net change in vegetation cover over the same period. Development of the tidal flat profile can be seen in the panels D & E which correspond to the two transects, depicted by black lines running across the tidal flat (D and E). Put in sequence, panels A - C support our explanation of marsh expansion, where (i) tidal flats rise and flatten as a consequence of higher rates of accretion at the seaward end of the tidal flat, (ii) micro-topographic patterns develop on the raised flattened anterior region, (iii) vegetation is able to expand to a greater extent over areas harboring micro-topographic pattens. Source data are provided as a Source Data file.

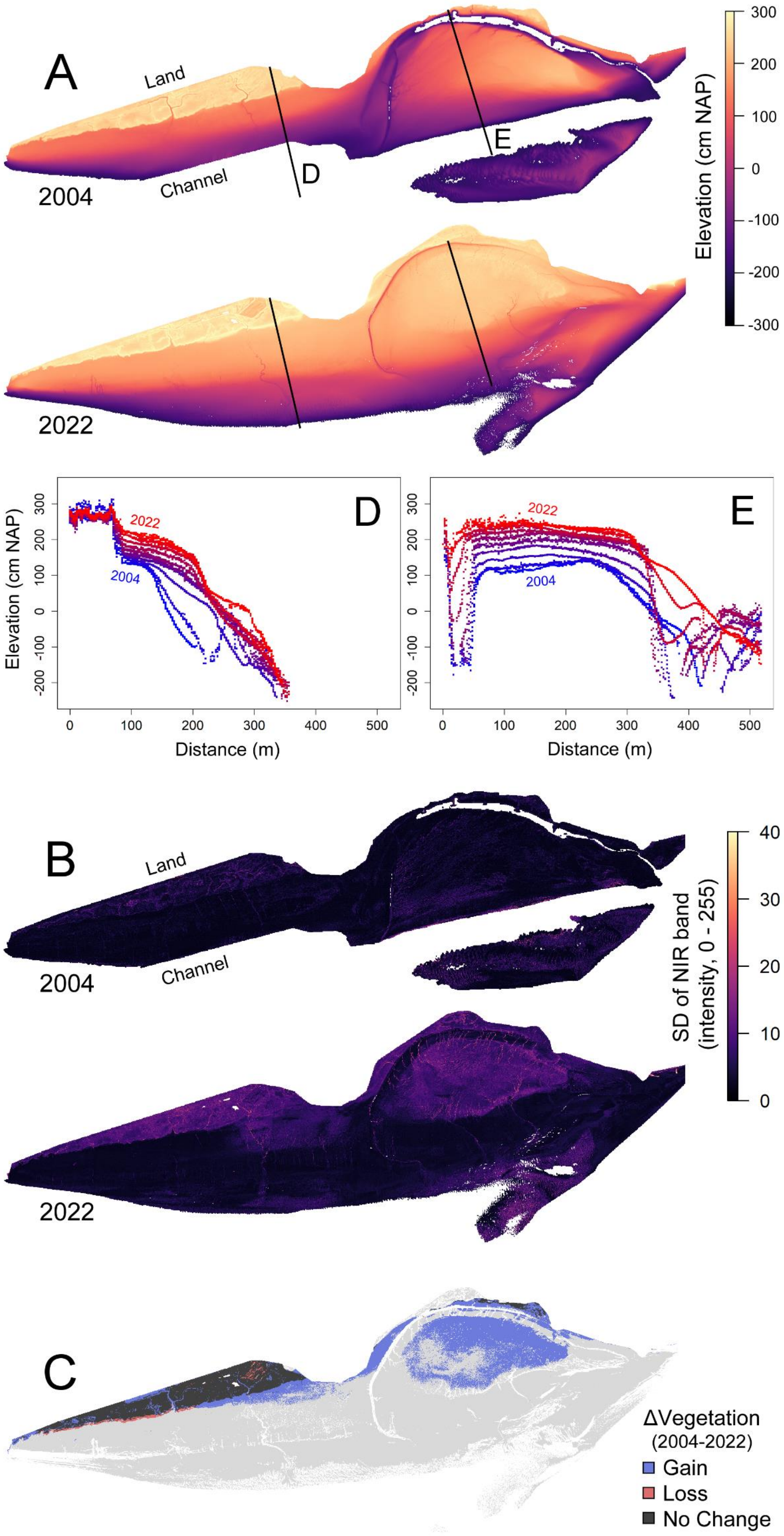

A
Land
Channel
D
E
2004
2022
Elevation (cm NAP)
300
200
100
0
-100
-200
-300
D
E
2022
2004
Elevation (cm NAP)
Distance (m)
0
100
200
300
400
500
-200
-100
B
Land
Channel
2004
2022
SD of NIR band
(intensity, 0 - 255)
40
30
20
10
0
C
ΔVegetation
(2004-2022)
Gain
Loss
No Change

Supplementary Figure 5. **The frequency of different NDVI values over the upper intertidal (above neap high water level) are displayed in four panels.** Each panel is calculated from an orthophotomosaic of one estuary in the indicated year. Dark coloration represents a high frequency of a specific NDVI value at a given intertidal position. NDVI values tend to fall into two distinct bands in the upper intertidal, indicating (1) vegetation at high NDVI values, and (2) sediment at lower values. The NDVI threshold that differentiates vegetation from sediment (indicated by the black line), falls halfway between these two bands. Due to differences in conditions during photography, the positions of the bands tend to vary between years. As such, these figures demonstrate the rationale behind recalculating a unique NDVI threshold for each estuary, in each year, as detailed in the methods. See supplementary appendix 2 for a complete list of the NDVI-thresholds used. Source data are provided as a Source Data file.

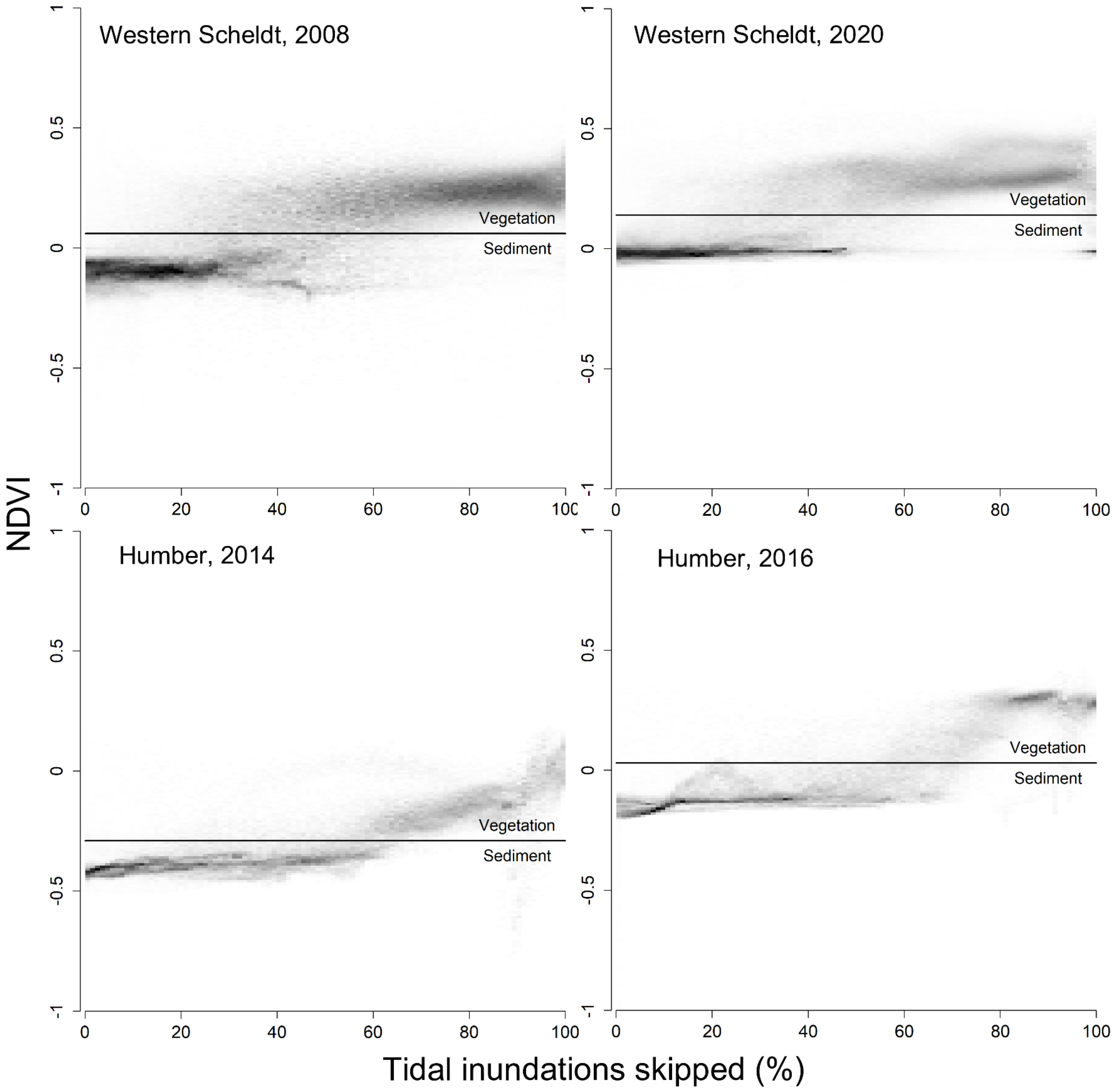
Western Scheldt, 2008
Western Scheldt, 2020
Humber, 2014
Humber, 2016
Vegetation
Sediment
NDVI
Tidal inundations skipped (%)
1
0.5
0
-0.5
-1
0
20
40
60
80
100

Supplementary Figure 6. **This figure showcases the utility of using orthophotos to quantify micro-topographic patterns, rather than relying on realtively coarser resolution lidar-derived elevation maps.** Here, the upper intertidal flat near the marsh, Groot Buitenschoor, in the Western Scheldt in 2020 is shown (coordinates: 51.375, 4.242). The slope of the tidal flat (left), calculated from aerial lidar data is displayed beside the standard deviation of the NIR band (right), calculated using falsecolor orthophotos. This second metric is used to indicate the intensity of micro-topographic patterns on tidal flats in this study (over a 2 m window). The small-scale structure of the micro-topographic pattern can be seen in the sub panel containing the original NIR image (12.5 cm resoltion). Due to the fine scale of these patterns they are invisible in coarser (2 m resolution) bathymetric slope map. Note that to aid visualization, the maps shown here are higher resolution than those used in the study anlysis (2 x 2 m instead of 10 x 10 m resolution).

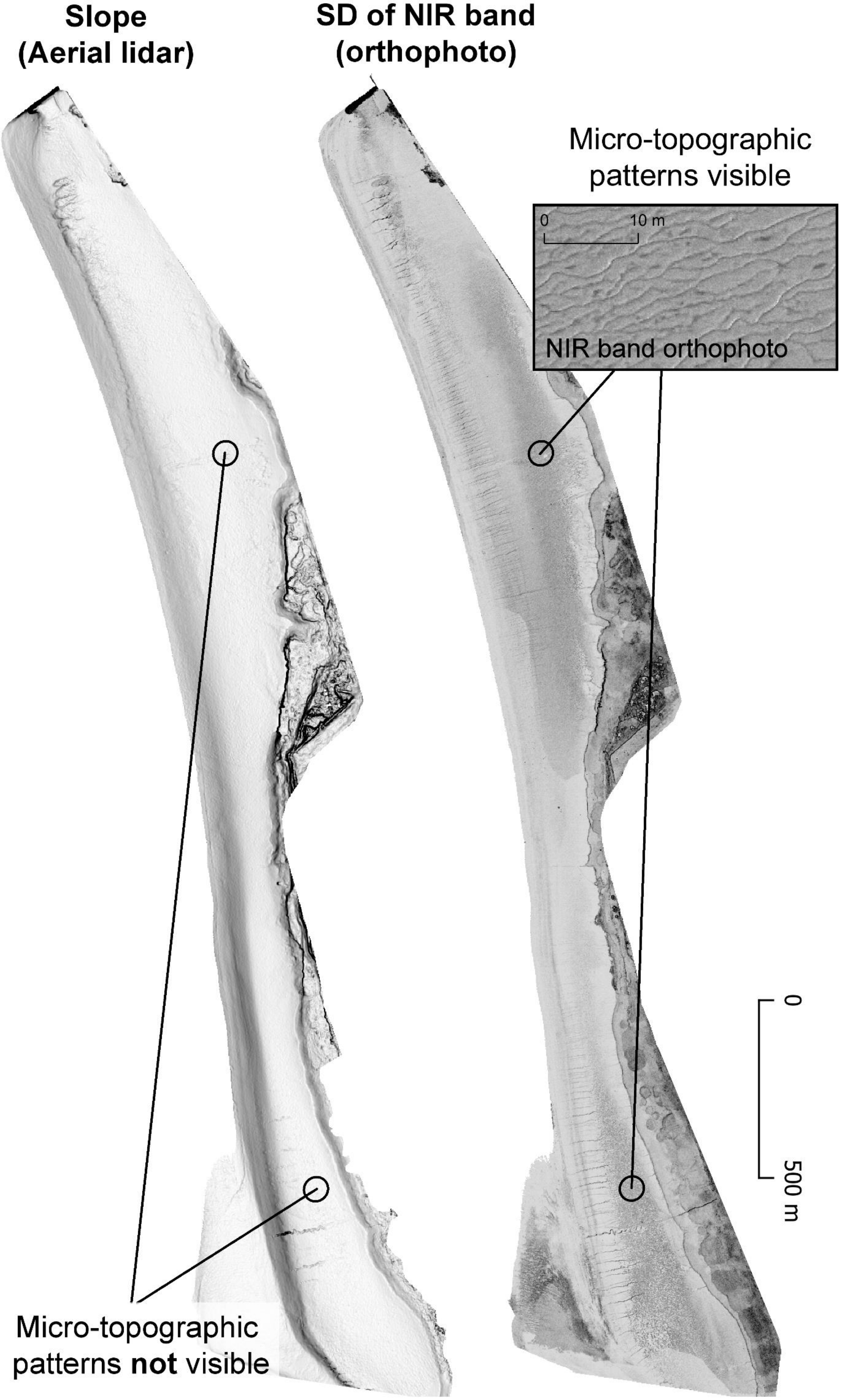
Slope
(Aerial lidar)
SD of NIR band
(orthophoto)
Micro-topographic
patterns visible
0
10 m
NIR band orthophoto
0
500 m
Micro-topographic
patterns not visible

Supplementary Figure 7. **A calibration of the technique of using photos to measure micro-topography, compared with elevation measurements using terrestrial lidar.** In panels A & B, the elevation and slope rasters produced by aerial lidar is compared with the higher resolution product of a terrestrial lidar survey (RIEGL VZ-400i, RIEGL Laser Measurement Systems GmbH, Horn, Austria). While the large-scale patterns in elevation change are captured equally in both methods (panel A), it is clear that the micro-topographic patterning is mostly averaged out in the coarser aerial lidar surveys (panel B). Terrestrial surveys are however not available for the entire estuary. Fortunately, the micro-topographic patterns detected in terrestrial surveys (panel B) are also visible in the estuary-wide NIR-band orthophotomosaics (panel C). In panel D, we compare the standard deviation of the slope captured by terrestrial lidar with the standard deviation of nir-band photos, measured over a 10 x 10m sampling grid, using a linear regression. A strong correlation between the two metrics can be seen. Source data are provided as a Source Data file.

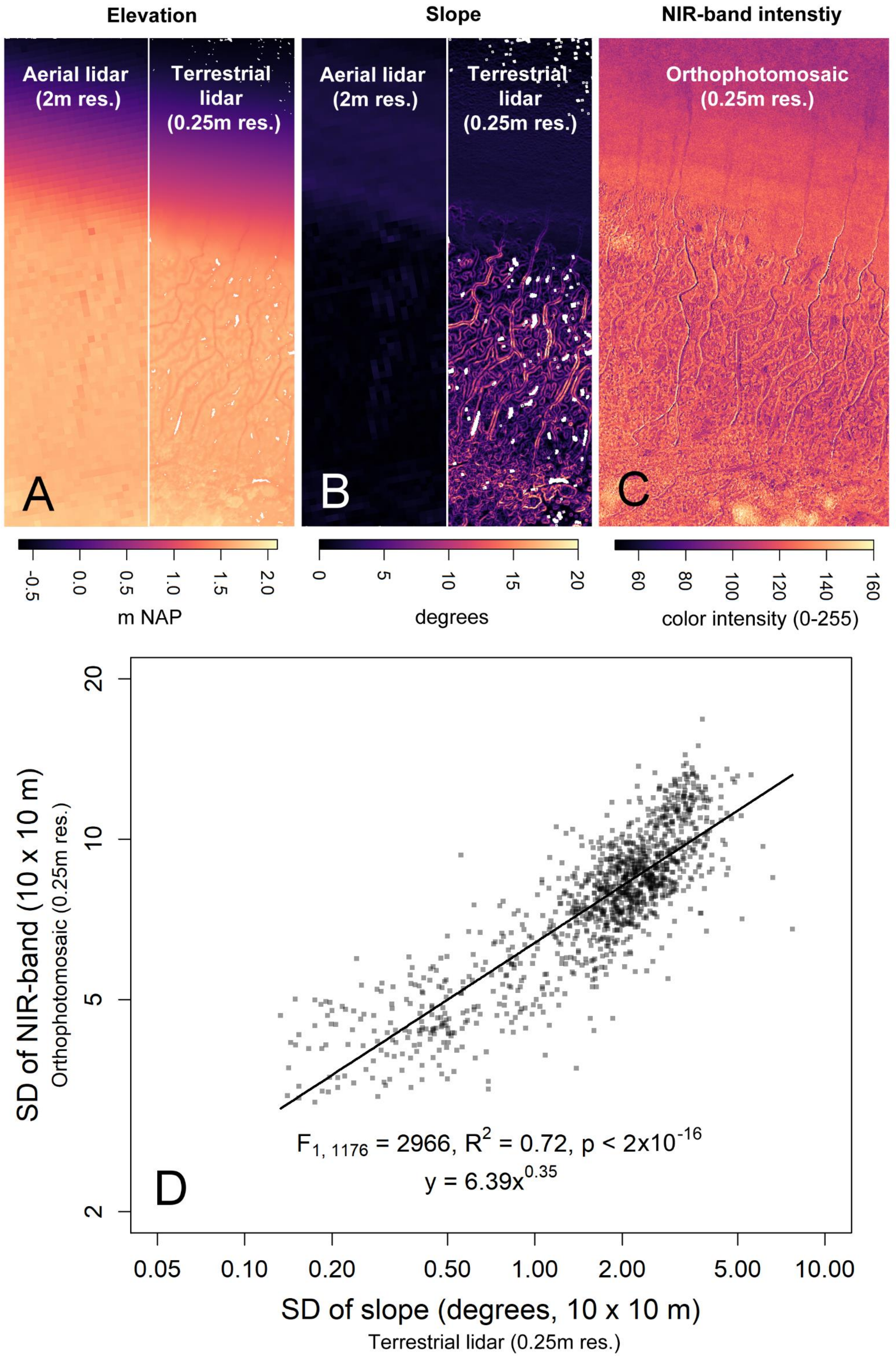

Elevation
Slope
NIR-band intenstiy
Aerial lidar (2m res.)
Terrestrial lidar (0.25m res.)
Aerial lidar (2m res.)
Terrestrial lidar (0.25m res.)
Orthophotomosaic (0.25m res.)
A
B
C
-0.5 0.0 0.5 1.0 1.5 2.0
m NAP
0 5 10 15 20
degrees
60 80 100 120 140 160
color intensity (0-255)
SD of NIR-band (10 x 10 m)
Orthophotomosaic (0.25m res.)
20
10
5
2
F1, 1176 = 2966, R2 = 0.72, p < 2x10-16
y = 6.39x0.35
D
0.05 0.10 0.20 0.50 1.00 2.00 5.00 10.00
SD of slope (degrees, 10 x 10 m)
Terrestrial lidar (0.25m res.)

Supplementary Figure 8. **This figure depicts the various regions of the estuary that are masked (removed) before the quantification of micro-topography.** The tidal flats displayed here are located near the marshes at Zuidgors and Baarland in the Western Scheldt, as photographed in 2020 (coordinates: 51.389, 3.857). Grey tones indicate the standard deviation in the NIR-band, measured in 2 x 2 m windows, the metric we used to indicate the intensity of micro-topography. In the upper intertidal, micro-topographic patterns can been seen as dark tones on the mud flat. However dark tones in the lower intertidal are caused by mega-ripples. Vegetation and tidal creeks in the upper intertidal also return a strong signal with this metric. For the SD of NIR metric to be indicative only of micro-topographic patterns, these other features must be excluded from the analysis. Therefore, vegetated areas (green), tidal creeks (red), and intertidal areas below neap high water level (blue-tinted), where mega ripples tend to form are all removed before the quantification of micro-topography. Note that to aid visualization of the structure of bathymetric patterns, the maps shown here are higher resolution than those used in the study anlysis (2 x2 m instead of 10x10 m resolution).

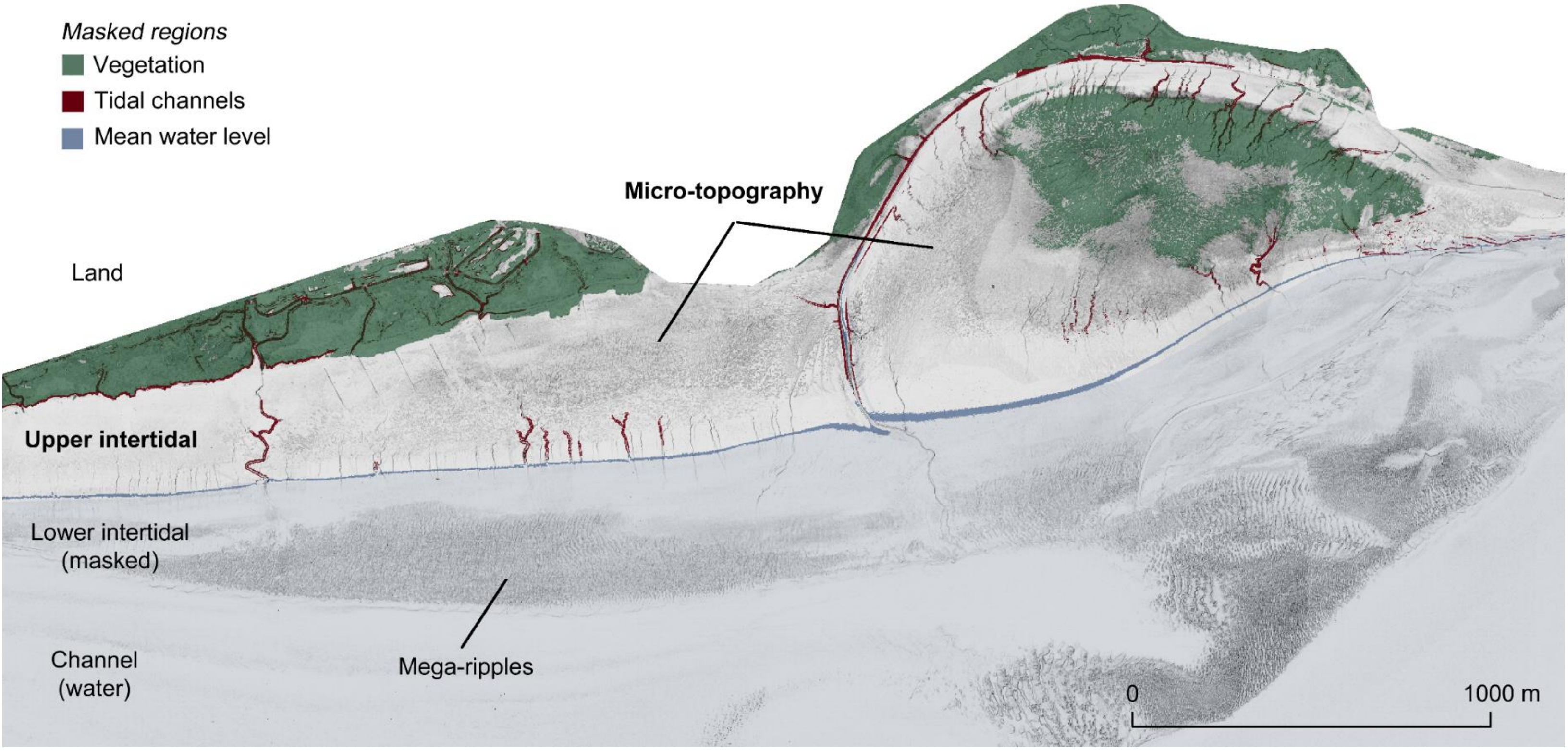

Masked regions
Vegetation
Tidal channels
Mean water level
Micro-topography
Land
Upper intertidal
Lower intertidal
(masked)
Channel
(water)
Mega-ripples
0
1000 m

Supplementary Figure 9. **An extended version of Figure 3.** In addition to the original figure (A), here we also display the total number of observations represented within each cell in the raster (B) and separate the effects of the tidal position (panel C) and the intensity of micro-topography (D) on vegetation establishment into 1D scatterplot figures. The points in panels C and D represent the mean value within each binned group, and the error bars show standard error (the exact number of replicate measurements within each binned group (n) for each mean can be found in the source data. Source data are provided as a Source Data file.

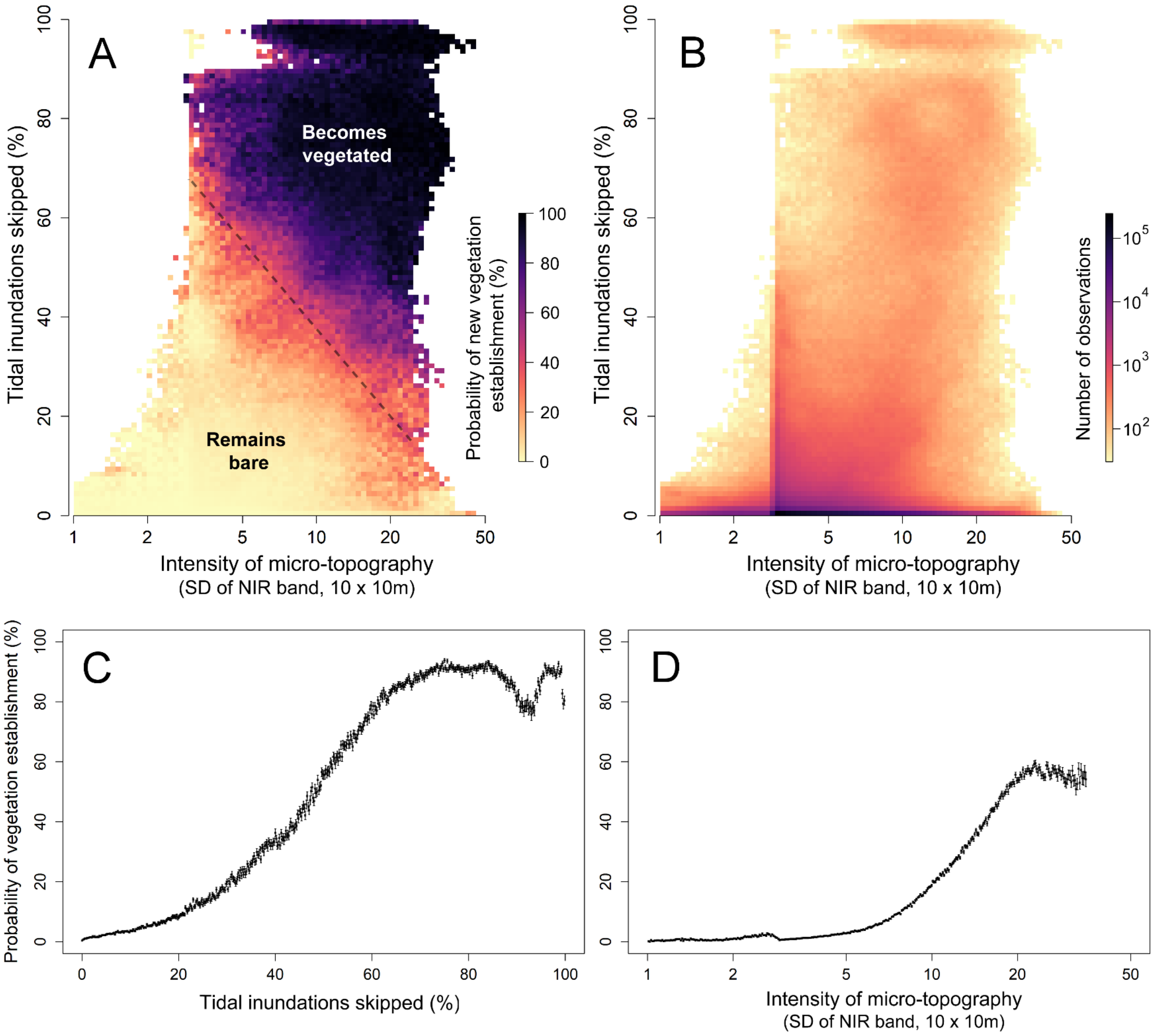

A
Becomes vegetated
Remains bare
Tidal inundations skipped (%)
Intensity of micro-topography
(SD of NIR band, 10 x 10m)
Probability of new vegetation establishment (%)
B
Tidal inundations skipped (%)
Intensity of micro-topography
(SD of NIR band, 10 x 10m)
Number of observations
C
Probability of vegetation establishment (%)
Tidal inundations skipped (%)
D
Intensity of micro-topography
(SD of NIR band, 10 x 10m)
