# Supplementary material for: Early indicators of tidal ecosystem shifts in estuaries
Source: Nat Commun. 2023 Apr 6;14:1911. doi: 10.1038/s41467-023-37444-6 (PMC10079839; doi:10.1038/s41467-023-37444-6)
Supplement: Supplementary file 3 — Reporting Summary [file 41467_2023_37444_MOESM3_ESM.pdf]

nature portfolio

Corresponding author(s): Gregory S. Fivash
Last updated by author(s): Dec 20, 2022

Reporting Summary

Nature Portfolio wishes to improve the reproducibility of the work that we publish. This form provides structure for consistency and transparency in reporting. For further information on Nature Portfolio policies, see our Editorial Policies and the Editorial Policy Checklist.

Statistics

For all statistical analyses, confirm that the following items are present in the figure legend, table legend, main text, or Methods section.

n/a	Confirmed	
☐	☒	The exact sample size (n) for each experimental group/condition, given as a discrete number and unit of measurement
☐	☒	A statement on whether measurements were taken from distinct samples or whether the same sample was measured repeatedly
☐	☒	The statistical test(s) used AND whether they are one- or two-sided<br>*Only common tests should be described solely by name; describe more complex techniques in the Methods section.*
☒	☐	A description of all covariates tested
☐	☒	A description of any assumptions or corrections, such as tests of normality and adjustment for multiple comparisons
☐	☒	A full description of the statistical parameters including central tendency (e.g. means) or other basic estimates (e.g. regression coefficient) AND variation (e.g. standard deviation) or associated estimates of uncertainty (e.g. confidence intervals)
☐	☒	For null hypothesis testing, the test statistic (e.g. F, t, r) with confidence intervals, effect sizes, degrees of freedom and P value noted<br>*Give P values as exact values whenever suitable.*
☒	☐	For Bayesian analysis, information on the choice of priors and Markov chain Monte Carlo settings
☒	☐	For hierarchical and complex designs, identification of the appropriate level for tests and full reporting of outcomes
☒	☐	Estimates of effect sizes (e.g. Cohen's d, Pearson's r), indicating how they were calculated

Our web collection on statistics for biologists contains articles on many of the points above.

Software and code

Policy information about availability of computer code

Data collection	No software was used
Data analysis	All data analysis was performed in R version 4.0.2. Many of our geospatial analyses depended on functions found in the R 'raster' library version 3.3-13. The 'inclined plane' numerical model was written in Python version 3.8.10. All scripts & code used in this study are publicly available via 4TU.Research (see Data & Code Availability sections).

For manuscripts utilizing custom algorithms or software that are central to the research but not yet described in published literature, software must be made available to editors and reviewers. We strongly encourage code deposition in a community repository (e.g. GitHub). See the Nature Portfolio guidelines for submitting code & software for further information.

Data

Policy information about availability of data

All manuscripts must include a data availability statement. This statement should provide the following information, where applicable:

- Accession codes, unique identifiers, or web links for publicly available datasets
- A description of any restrictions on data availability
- For clinical datasets or third party data, please ensure that the statement adheres to our policy

The geospatial data used in the analyses that support the findings of this study are archived and publicly available via 4TU.Research Data, doi:10.4121/21762680. Source data for figures 2 - 4, and supplementary figures 1-5, 7, and 9 are provided with the paper.

Human research participants

Policy information about studies involving human research participants and Sex and Gender in Research.

Reporting on sex and gender	No human research participants were involved in this study.
Population characteristics	No human research participants were involved in this study.
Recruitment	No human research participants were involved in this study.
Ethics oversight	No human research participants were involved in this study.

Note that full information on the approval of the study protocol must also be provided in the manuscript.

Field-specific reporting

Please select the one below that is the best fit for your research. If you are not sure, read the appropriate sections before making your selection.

☐ Life sciences ☐ Behavioural & social sciences ☒ Ecological, evolutionary & environmental sciences

For a reference copy of the document with all sections, see nature.com/documents/nr-reporting-summary-flat.pdf

Ecological, evolutionary & environmental sciences study design

All studies must disclose on these points even when the disclosure is negative.

Study description	This was a geospatial study, exploring the development of the morphology and vegetation cover of three European estuaries over the last 15-20 years (the period for which high-quality data is available). Statistical tests were limited to linear regressions fit to time series to quantify rates of change, calculations of means and standard errors, and in one case a linear regression was used to quantify the correlation between the 'intensity of micro-topography' and tidal inundations skipped (%), which was performed after log-transforming both variables. The transformations were performed because the correlation between the variables was best explained by a power-law, as suggested by the AIC, when compared to linear models that did not include transformed variables.
Research sample	Geospatial data included publicly-available false color orthophotomosaics and aerial lidar, provided by Dutch, British, and Germany governmental bodies (NL: Rijkswaterstaat, UK: Defra Survey, DE: German Federal Waterways and Shipping administration (WSV)). False color images were used to classify the presence of vegetation and the presence of micro-topographic patterns on tidal flats, which were not detected in coarser (2m resolution) digital elevation models (DEMs). DEMs produced through aerial lidar were used to determine the elevation and slope of the tidal flat. Terrestrial lidar data was also collected on-site by Gregory S. Fivash and Jeroen van Dalen of the Netherlands Institute for Sea Research (NIOZ) in Yerseke. This data was used to calibrate the relationship between measured bathymetric micro-topography (measured by the terrestrial lidar), and the indirect measure of micro-topography using orthophotos. This measurement produces a x-y-z point cloud, which was rasterized using the arcGIS LAS to Raster tool. The raster resolution chosen was equal to that of the publicly provided orthophotomosaics to ease comparison.
Sampling strategy	We attempted to use all of the data that was publicly available. Our geospatial analyses were performed at the scale of the entire estuary of focus, instead of on specific field sites in order to maximize our sampling size. Our sample unit was a 10 x 10m grid cell, which was determined by the spatial scale of our coarsest measurement, the 'intensity of micro-topography' metric (SD of NIR-band in orthophotos). All other measurements were aggregated to this spatial scale to allow proper comparison between measured quantities.
Data collection	Data collection was commissioned by the respective governmental bodies and provided via online web portals:<br>- Dutch Ministry of Infrastructure and Water Management (Rijkswaterstaat), available at https://www.rijkswaterstaat.nl/apps/geoservices/geodata/dmc/.<br>- German Federal Waterways and Shipping administration (WSV, available at https://www.kuestendaten.de/DE/Services/Kartenthemen/Kartenthemen_node.htm)<br>- British Department of Environment Food & Rural Affairs (via the Defra Survey Data Download portal, https://environment.data.gov.uk/DefraDataDownload/?Mode=survey).<br><br>Terrestrial lidar data was measured and recorded internally by a RIEGL VZ-400i (RIEGL Laser MeasurementSystems GmbH, Horn, Austria). Terrestrial lidar surveys were performed by Gregory S. Fivash and Jeroen van Dalen of the Netherlands Institute for Sea Research (NIOZ) in Yerseke. These surveys included 4 scans of a 300 m circular plot from each cardinal direction, that were combined to create a single point cloud, and then rasterized to a 0.25 x 0.25 m grid.
Timing and spatial scale	The timing and spatial scale of publicly available geospatial data was determined by the respective governmental bodies (see above).<br>-For the Western Scheldt, data was available between 2004-2020<br>-For the Elbe, DEMs were available between 2006-2016, and orthophotos were available between 2002-2016.<br>-For the Humber, data was available between 2002-2019<br><br>Repeated terrestrial lidar surveys were performed by G.S. Fivash & J. van Dalen every three months between Fall 2020 and Fall 2022

	across 3 sites selected for the presence of tidal flat micro-topography. Of these surveys, only those which occurred nearest in time to a (semi-)annual national lidar/orthophoto survey were used in the calibration. The 3 field sites were chosen due to the presence of micro-topographic patterns. Each measurement contained four overlapping laser scans (covering in total a circular region with a diameter of roughly 300 m). This spatial scale was chosen to be as large as possible given constraints on time allowed in the field.
Data exclusions	During the analysis of micro-topographic patterns, vegetation (determined by NDVI), creeks (determined by slope) and other bed forms (determined by position in the intertidal frame), were excluded so that the metric used to quantify micro-topographic patterns (SD of NIR-band in orthophotos) did not give a false signal given by these other complex features.<br><br>In Figures 3 & 4, and supplementary figures 3 & 9, data was binned into groups to identify correlations between variables that were not specifically linear, or quantified by any particular fitted curve. When bins included less than 30 measurements, that binned group was excluded due to the untrustworthiness of the estimate it provided. In the 2D raster plots, these poorly sampled regions are indicated by a white background color.
Reproducibility	No manipulative experiments were performed in this study. Our analyses of the publicly available data can be reproduced using the publicly-available scripts and datasets we have provided (see Data & Code Availability section of manuscript).
Randomization	Our study did not involve grouping in a randomized trial.
Blinding	Blinding was not possible in this study since it did not involve experimental trials, only the analysis of publicly available data.

Did the study involve field work? ☒ Yes ☐ No

Field work, collection and transport

Field conditions	Terrestrial laser scanning of tidal flat field sites during low tide, repeated at 3 month intervals for 2 years between 2020-2022. This data was used as a ground-truthing test to verify the accuracy of quantifying micro-topographic pattern intensity using orthophotos.
Location	Three sites in the Western Scheldt esutary, the Netherlands: Hoofdplaat (51.372805, 3.676586), Zuidgors (51.390810, 3.855634), Baarland (51.393110, 3.866620).
Access & import/export	The Royal Netherlands Institute for Sea Research (NIOZ) has been granted formal written permission by the land owners to access these field sites, renewed annually. These land owners include the organizations Natuurmonumenten (2022-01-01) & Het Zeeuwse Landschap (2021-12-09).
Disturbance	This measurement itself is non-invasive. Foot prints left in the mud by fieldworkers walking between measuring positions usually disappear within a few weeks. Fieldworkers walk in a designated path to minimize disturbance.

Reporting for specific materials, systems and methods

We require information from authors about some types of materials, experimental systems and methods used in many studies. Here, indicate whether each material, system or method listed is relevant to your study. If you are not sure if a list item applies to your research, read the appropriate section before selecting a response.

Materials & experimental systems

n/a	Involved in the study
☒	☐ Antibodies
☒	☐ Eukaryotic cell lines
☒	☐ Palaeontology and archaeology
☒	☐ Animals and other organisms
☒	☐ Clinical data
☒	☐ Dual use research of concern

Methods

n/a	Involved in the study
☒	☐ ChIP-seq
☒	☐ Flow cytometry
☒	☐ MRI-based neuroimaging
